# Biomarkers in pulmonary infections: a clinical approach

**DOI:** 10.1186/s13613-024-01323-0

**Published:** 2024-07-17

**Authors:** Pedro Póvoa, Luís Coelho, José Pedro Cidade, Adrian Ceccato, Andrew Conway Morris, Jorge Salluh, Vandack Nobre, Saad Nseir, Ignacio Martin-Loeches, Thiago Lisboa, Paula Ramirez, Anahita Rouzé, Daniel A. Sweeney, Andre C. Kalil

**Affiliations:** 1 Department of Intensive Care, Hospital de São Francisco Xavier, ULSLO, Lisbon, Portugal; 2 NOVA Medical School, Faculdade de Ciências Médicas, Universidade Nova de Lisboa, Campo dos Mártires da Pátria 130, 1169-056 Lisbon, Portugal; 3 Center for Clinical Epidemiology and Research Unit of Clinical Epidemiology, OUH Odense University Hospital, Odense, Denmark; 4 Pulmonary Department, CDP Dr. Ribeiro Sanches, ULS Santa Maria, Lisbon, Portugal; 5 Critical Care Center, Institut d’Investigació i Innovació Parc Taulí I3PT-CERCA, Hospital Universitari Parc Taulí, Univeristat Autonoma de Barcelona, Sabadell, Spain; 6 CIBER of Respiratory Diseases (CIBERES), Institute of Health Carlos III, Madrid, Spain; 7 Intensive Care Unit, Hospital Universitari Sagrat Cor, Grupo Quironsalud, Barcelona, Spain; 8 Division of Anaesthesia, Department of Medicine, University of Cambridge, Cambridge, UK; 9 Division of Immunology, Department of Pathology, University of Cambridge, Cambridge, UK; 10 JVF Intensive Care Unit, Addenbrooke’s Hospital, Cambridge, UK; 11 Postgraduate Program, D’Or Institute for Research and Education (IDOR), Rio de Janeiro, Brazil; 12 Postgraduate Program of Internal Medicine, Federal University of Rio de Janeiro, (UFRJ), Rio de Janeiro, Brazil; 13 School of Medicine, Universidade Federal de Minas Gerais, Belo Horizonte, Brazil; 14 1Univ. Lille, UMR 8576-UGSF-Unité de Glycobiologie Structurale et Fonctionnelle, 59000 Lille, France; 15 CNRS, UMR 8576, 59000 Lille, France; 16 INSERM, U1285, 59000 Lille, France; 17 CHU Lille, Service de Médecine Intensive Réanimation, 59000 Lille, France; 18 Department of Intensive Care Medicine, Multidisciplinary Intensive Care Research Organization (MICRO), St. James Hospital, Dublin, Ireland; 19 Department of Pneumology, Hospital Clinic of Barcelona—August Pi i Sunyer Biomedical Research Institute (IDIBAPS), University of Barcelona, Barcelona, Spain; 20 Postgraduate Program Pulmonary Science, Hospital de Clínicas de Porto Alegre, Universidade Federal do Rio Grande do Sul, Porto Alegre, Rio Grande do Sul Brazil; 21 Department of Critical Care Medicine, Hospital Universitario Y Politécnico La Fe, Valencia, Spain; 22 Division of Pulmonary, Critical Care and Sleep Medicine, Department of Medicine, University of California, La Jolla, San Diego, CA USA; 23 Department of Internal Medicine, Division of Infectious Diseases, University of Nebraska Medical Center, Omaha, NE USA

**Keywords:** Pulmonary infections, Pathogen-specific biomarkers, Host-response biomarkers, C-reactive protein, Procalcitonin

## Abstract

Severe acute respiratory infections, such as community-acquired pneumonia, hospital-acquired pneumonia, and ventilator-associated pneumonia, constitute frequent and lethal pulmonary infections in the intensive care unit (ICU). Despite optimal management with early appropriate empiric antimicrobial therapy and adequate supportive care, mortality remains high, in part attributable to the aging, growing number of comorbidities, and rising rates of multidrug resistance pathogens. Biomarkers have the potential to offer additional information that may further improve the management and outcome of pulmonary infections. Available pathogen-specific biomarkers, for example, *Streptococcus pneumoniae* urinary antigen test and galactomannan, can be helpful in the microbiologic diagnosis of pulmonary infection in ICU patients, improving the timing and appropriateness of empiric antimicrobial therapy since these tests have a short turnaround time in comparison to classic microbiology. On the other hand, host-response biomarkers, for example, C-reactive protein and procalcitonin, used in conjunction with the clinical data, may be useful in the diagnosis and prediction of pulmonary infections, monitoring the response to treatment, and guiding duration of antimicrobial therapy. The assessment of serial measurements overtime, kinetics of biomarkers, is more informative than a single value. The appropriate utilization of accurate pathogen-specific and host-response biomarkers may benefit clinical decision-making at the bedside and optimize antimicrobial stewardship.

**Supplementary Information:**

The online version contains supplementary material available at 10.1186/s13613-024-01323-0.

## Background

The management of severe acute respiratory infections remains a major challenge for those caring for critically ill patients. Community-acquired pneumonia (CAP) is one of the most frequent causes of admission to the intensive care unit (ICU) worldwide, while hospital-acquired and ventilator-associated pneumonia (HAP and VAP) are among the most frequent and lethal pulmonary infections in the ICU [1–3]. Despite the availability of efficient broad-spectrum antimicrobials, mortality rates remain elevated, which has been attributed at least in part to the aging and comorbidities of the population [4], rising rates of multi-resistant pathogens [5], and adverse events associated with the treatment use of supportive care and antimicrobials [6–9].

Therefore, strategies have been proposed to help guide the duration of antimicrobial therapy by ensuring both its appropriate use to achieve clinical cure and avoid excessive drug exposure, thus mitigating the above-mentioned problems and the impact on microbiota [10]. In this scenario, protein-based biomarkers, both pathogen-specific and host-response biomarkers have been evaluated [11–13] to help clinicians optimize antimicrobial stewardship at a patient level [12]. In this review, the potential role of omics as well as molecular diagnostic tests in severe respiratory infections will not be addressed.

In the present narrative review, we will revise the current literature and provide a clinical approach for the optimal use of biomarkers in the management of pulmonary infections of immunocompetent critically ill patients.

## Pathogen-specific biomarkers

Following diagnostic suspicion of pulmonary infection, based on clinical manifestations and radiologic findings, the next step is selection of an appropriate empiric antimicrobial agent(s) based on the most likely causative pathogen(s) [14, 15]. Fear of overlooking a potential pathogen or resistance mechanism, and this has led to the widespread practice of prescribing unnecessary broad-spectrum antimicrobials [16]. Unfortunately, the turnaround time of traditional microbiology culture results is at least 2–3 days. Having access to other tests, namely pathogen-specific biomarkers, capable of identifying specific pathogens in a couple of hours could potentially prove invaluable in better targeting empiric therapy [12, 15].

Influenza and COVID-19 epidemics have resulted in significant morbidity and mortality worldwide. Their diagnosis is made on clinical grounds, laboratory testing, radiologic findings, and local epidemiology information. Several diagnostic tests with variable sensitivities and specificities are currently available in clinical settings, including antigen detection immunoassays, and molecular assays (nucleic acid detection) that utilize respiratory tract specimens. Rapid antigen tests are straightforward to perform and take a short time to complete (< 15 min). The specificity of rapid antigen tests is high, but sensitivity is low; additionally, false‐positive results may occur due to low infection activity or the presence of non-viable pathogens [17, 18].

The pneumococcal urinary antigen test detects the C-polysaccharide antigen produced by *Streptococcus pneumoniae*. This test demonstrates a sensitivity range of 50 to 80% and a specificity exceeding 90% [19]. It is worth noting that the results from this test are typically available within approximately 30 min. Moreover, it has been rigorously validated for both urine and cerebrospinal fluid. The clinical significance of this test cannot be understated, as *Streptococcus pneumoniae* is the most frequently encountered culprit in cases of CAP with identified bacterial etiology.

A positive urinary pneumococcal antigen test strongly suggests a pneumococcal infection, most commonly pneumonia [20]. However, it is important to acknowledge that a negative result cannot conclusively rule out a pneumococcal infection. Urinary pneumococcal antigen test may yield positive results in approximately 50% of patients with pneumococcal pneumonia during the month following diagnosis or even beyond [21]. False positives can also occur, especially in individuals receiving the streptococcus pneumoniae vaccine within five days prior to the test [20]. Although the urinary antigen test is recommended for severe CAP patients, its impact on clinical outcomes seems somewhat limited, including the possibility of narrowing the spectrum of antibiotic therapy [20, 22].

Legionella urinary antigen testing is also available, this specifically targets *Legionella pneumophila* serogroup 1-soluble antigen and sensitivity ranges between 70 to 100%, with specificity reportedly 95 to 100% [23]. It is important to note that *Legionella* is an infrequent pathogen in CAP, typically associated with outbreaks [24] or recent travel. *Legionella pneumophila* serogroup 1 is responsible for around 80% of reported cases of Legionellosis. Due to the inherent challenge in culturing this pathogen, the presence of Legionella antigen in urine is very useful. Typically, this antigen can be detected in urine as early as three days after the onset of symptoms [23]. Consequently, a positive test result justifies the modification of antibiotic therapy. A negative test result suggests the absence of a recent or current Legionella infection or a strain other than serogroup-1. Nevertheless, in the initial stages of infection, the antigen may not be detectable in the urine, and the involvement of other *Legionella pneumophila* serogroups and other Legionella species cannot be entirely ruled out [12].

Cryptococcal glucuronoxylomannan antigen (CRAG) testing is the only commercially available biomarker to detect Cryptococcal infections and its role in identifying pneumonia is limited. Cryptococcal pulmonary disease can be categorized in terms of three human host populations: patients with HIV; patients who are organ transplant recipients (OTR); and patients who neither have HIV nor are OTRs, many of whom, however, have a compromised cell-mediated immunity (Table 1) [25]. The greater the disease burden or the presence of disseminated disease, the more likely serum CRAG testing will identify infection. CRAG testing is almost always positive in patients with HIV (in particular, patients with AIDS) who have cryptococcal pneumonia as they will also typically have disseminated disease, importantly this should prompt CSF testing for meningoencephalitis. Based on limited data, serum CRAG for the diagnosis of cryptococcal pneumonia amongst patients without HIV appears to be less useful, especially in patients who do not have disseminated disease or who are not OTRs. Among patients without HIV, CRAG testing of BAL fluid in conjunction with serum CRAG testing has been shown in one study, to improve overall diagnostic sensitivity [26].

**Table 1:** Pathogen-specific biomarkers used in the diagnosis of pulmonary infections

Biomarker	Methods	Turnaround time	Infection diagnosis	Sample	Diagnostic accuracy		Comments
					Sensitivity	Specificity	
Influenza A/B Ag test (83)	Immunoassay EIA ICT FIA	< 15 min	Influenza pneumonia	Nasal swab other respiratory samples	50–70%	90–95%	Qualitative results (positive vs negative) Sensitivity to detect influenza B < than for influenza A Accuracy varies according to prevalence of influenza (false positive more likely if prevalence is low and false negatives if prevalence is high)
SARS-CoV-2 Ag test (84)	Immunoassay ICT FIA	< 15 min	SARS-CoV-2 pneumonia	Nasal swab other respiratory samples	66–73%	99.2–99.3%	Need to follow the manufacturer’s instructions for use High rate of false negative if tested before symptom onset False positive are unlikely (unless low prevalence)
*Streptococcus pneumoniae* urinary Ag test	Immunoassay ICT FIA	15–30 min	Pneumococcal pneumonia	Urine CSF	70–82%	93–99%	Specificity is lower in paediatric population consequence of nasopharyngeal colonisation with pneumococcus able to diagnose pneumococcal infection regardless of serotype; but lower sensitivity for the serotypes not included in the vaccine False positives: prior vaccination (48 h), previous infection (several months)
*Legionella* urinary Ag test	Immunoassay EIA ICT FIA	15-90 min	Legionellosis caused by *Legionella* spp.	Urine	70–100%	95–100%	Only for *Legionella pneumophila* serogroup 1 (LP1) that accounts for 84% of cases Test remain positive for a few weeks after infection negative result could have disease caused by a non LP1
Cryptococcal glucuronoxylomannan Ag test (85)	Immunoassay EIA LFA Latex agglutination	LFA < 10 min CRAG Latex 4 h	**HIV** Cryptococcal pulmonary disease	Serum BAL	70% 40–80%	99% 98%	Detects all cryptococcal serotypes LFA is more sensitive to lower antigen levels
			**OTR** Cryptococcal pulmonary disease	serum	73–100%		
			**No HIV, no OTR** Cryptococcal pulmonary disease	Serum BAL	40–92% 82.6–100%		
Histoplasma Ag test (86)	Immunoassay EIA LFA	LFA 40 min Other hours		Urine Serum BAL	79% 82% 94%	99% 97% 98%	Sensitivity of the urine test varies according to being acute (83%), subacute (30.4%), chronic (87.5%) or disseminated (91.8%) disease Antigen testing alone cannot be used to rule out pulmonary Histoplasmosis cross reactivity with Blastomyces is reported to be 93–96%
(1,3)-β-D-glucan (BDG) (Fungitell® assay) (87)	Protease zymogen-based colorimetric assay	60 min	*Pneumocystis jirovecii* pneumonia	Serum	85–91%	75–87%	Specificity can improve by requiring two consecutive positive results higher BDG levels (> 200 pg/mL, Fungitell assay) are associated with clinically significant PJP in patients with positive PCR results a negative BDG effectively rules out PJP in situations with low to intermediate disease likelihood (≤ 20% in non-HIV patients) A negative BDG cannot rule out the diagnosis among patients with a higher likelihood of *Pneumocystis jirovecii* pneumonia
Galactomannan (GM) (88)	imMunoassay EIA	< 4 h	Invasive pulmonary aspergillosis	Serum (ODI ≥ 0.5) BAL (ODI ≥ 0.5)	30–100% 65–88%	61–100% 75–90%	Increasing cut-off (ODI ≥ 1) increases specificity but decreases sensitivity False negatives are frequent in non-neutropenic False positive tests have been reported in association with administration of certain antibiotics and cross reactivity exists with other fungal infections, such as those due to Fusarium spp. or Histoplasma capsulatum BAL GM is more useful than serum GN for diagnosis of invasive pulmonary aspergillosis
	LFA	20 min		Serum BAL	83% 90%	91% 92%	
lipoarabinomannan (LAM) test (Alere LAM) (89)	Immunoassay LFA	30 min	Tuberculosis	urine	52–56%	87–92%	WHO strongly recommends using urinary LAM testing in HIV-infected (specially with CD4 < 200 cells/mm3)

*Ag* antigen, *BAL* bronchoalveolar lavage, *BDG* (1,3)-β-D-glucan, *CRAG* Cryptococcal glucuronoxylomannan antigen, *EIA* enzyme immunoassay, *FIA* fluorescence immunoassay, *GM* galactomannan, *HIV* human immunodeficiency virus, *ICT* immunochromatographic test, *LFA* lateral flow assay, *ODI* optical density index, *OTR* organ transplant recipient, *WHO* World Health Organization

Antigen testing alone cannot be used to rule out pulmonary Histoplasmosis. As with Cryptococcal pneumonia, the sensitivity of antigen testing for pulmonary Histoplasmosis is related to both the patient’s immune status and the burden of disease. Again, the greatest sensitivity is found amongst the immunocompromised and when disseminated disease is present (Table 1) [27]. Histoplasma antigen testing for fungal pneumonia can be performed on urine, serum, or BAL fluid. Urine antigen testing tends to be more sensitive than serum antigen testing, particularly among patients with disseminated disease; nonetheless, overall testing sensitivity is highest when performed on both serum and urine [28]. BAL antigen testing, on the other hand, demonstrated superior sensitivity compared to urine-based testing in one study conducted among patients suspected of pneumonia most of whom had cell mediated immunodeficiency including HIV [29]. It should also be noted that cross-reactivity with Blastomyces is reported to be 93–96% as the two fungi share galactomannan cell wall antigens [30].

Coccidioidomycosis is endemic to the western hemisphere with most reported cases occurring in the US state of Arizona and southern California [31]. While some cases of reactivation have been described among the immunocompromised, in general, testing should be limited to immunocompetent individuals who either reside or have traveled (within 30 days of symptoms) to endemic areas [32]. Serologic testing is the preferred method of making a timely diagnosis. Testing with enzyme immunoassays (EIAs) is typically the initial step with confirmatory complement fixation and immunodiffusion tests performed at reference laboratories [33]. Early in the disease course, serologic testing may be falsely negative. Alternatively, IgM EIA testing may be falsely positive. As a result, serial testing is advised. In one landmark study, EIA testing showed 87% and 67% sensitivity among immunocompetent and immunosuppressed patients with pulmonary coccidioidomycosis but improved to 95% and 84% respectively when sequential and confirmatory testing was performed [34].

Serum (1,3)-β-D-glucan (BDG) is widely included in the diagnostic work-up for *Pneumocystis jirovecii* pneumonia (PJP) in immunocompromised patients. Proven PJP, diagnosed by microscopic detection of *P. jirovecii* cysts in respiratory specimens through conventional or immunofluorescence staining, is reported in less than one-third of cases, as observed in a recent international retrospective study including 600 critically ill patients suspected of PJP [35]. Otherwise, PJP diagnosis relies on a combination of (i) host factors—notably altered T-cell immunity, including steroids and CD4 lymphocyte counts < 200/µL induced by various underlying diseases, (ii) consistent radiological patterns—typically bilateral ground-glass infiltrates on chest computed tomography, and (iii) positive mycological tests, including Pneumocystis quantitative PCR in respiratory secretions and/or serum BDG [36, 37].

In a recent meta-analysis involving 997 patients diagnosed with PJP and 3062 controls, the pooled sensitivity of BDG for PJP diagnosis was relatively high (91%), especially in patients with HIV infection, but specificity was low (79%) [38]. Quite similar diagnostic performances were observed among ICU patients [35] (Table 1). Specificity can improve by requiring two consecutive positive results, to rule out false positives. Given its pan-fungal polysaccharide nature, BDG may also detect other fungal infections, that need to be excluded [36]. In practice, a negative BDG effectively rules out PJP in situations with low to intermediate disease likelihood (≤ 20% in non-HIV patients) [38]. Conversely, a positive BDG alone, at the manufacturer's recommended cut-off, is insufficient to diagnose PJP. Detecting *P. jirovecii* by qPCR in any respiratory specimen, including oral wash samples from the upper respiratory tract in non-intubated patients, is accepted for diagnosing PJP [36, 39]. However, a concurrently positive BDG proves valuable to differentiate between Pneumocystis colonization and infection in the presence of a positive qPCR, especially with a low fungal load [35]. Interestingly, higher BDG levels (> 200 pg/mL, Fungitell assay) are associated with clinically significant PJP in patients with positive PCR results [40]. Lastly, BDG lacks utility in monitoring treatment response or carrying specific prognostic value [41].

Galactomannan, a polysaccharide antigen primarily found in the cell walls of Aspergillus species, is a valuable biomarker for the early detection and monitoring of invasive fungal infections. Enzyme immunoassays (EIAs) or lateral flow assays are commonly used to detect galactomannan antigens in serum or BAL fluid samples. Traditionally, serum determinations have been valuable for immunocompromised individuals like transplant recipients and patients with hematologic malignancies [42]. Galactomannan testing aids clinicians in diagnosing invasive pulmonary aspergillosis, assisting in distinguishing between colonization and active infection and guiding appropriate antifungal therapy. However, the sensitivity and specificity of serum galactomannan testing vary across different patient populations and clinical settings, ranging from 30 to 100% for sensitivity and 61 to 100% for specificity. This variability is influenced by factors such as underlying diseases, sample types, and confounding factors, including the degree of angioinvasion, which is more common in severely immunocompromised patients [43]. A galactomannan optical density index (ODI) cutoff of ≥ 0.5 is typically used for serum samples, although cutoff values may differ for other sample types. Patients with respiratory infections caused by viruses such as SARS-CoV-2 or influenza can develop invasive pulmonary aspergillosis. In such cases, BAL fluid is the preferred sample for galactomannan testing due to the lower degree of angioinvasion observed in these patients [44]. For BAL diagnosis of invasive pulmonary aspergillosis an ODI of 1.0 is commonly used. False positives can occur with concurrent penicillins (most notably piperacillin/tazobactam and amoxicillin/clavulanic acid), recent intravenous immunoglobulin therapy and some dietary sources [45].

The traditional diagnostic methods of tuberculosis (TB), including sputum smear microscopy and culture, are very slow, time-consuming and have limitations, especially in cases of paucibacillary or extrapulmonary TB. There are a wide range of TB biomarkers specific either to the host or the pathogen. The most studied pathogen-specific biomarkers are the urine lipoarabinomannan (LAM) test (AlereLAM) [46]. Lipoarabinomannan is a component of the mycobacterial cell wall released from metabolically active bacterial cells and excreted in host urine. Although, being less expensive and a highly specific antigen that can be detected quickly, its low sensitivity in individuals other than severely immunocompromised HIV-positive patients remains a limitation. For these reasons, WHO recommends the use LAM only in HIV-positive patients who are severely ill or with a CD4 count lower than 100 cells/mm3 [47]. More recently, the development of Fujifilm SILVAMP TB LAM (FujiLAM) which detects urine LAM concentrations 30 times lower than AlereLAM allowed for improved sensitivity for TB detection in HIV-negative individuals and HIV-positive with higher CD4 counts. However, although promising, this new test still presents limitations that preclude their wider use [48].

## Host-response biomarkers

A host-response biomarker is any molecule produced by the host in response to any inflammatory insult that can be measured in the body and is related to that pathological process, namely an infection. Therefore, if an infection is the underlying inflammatory insult, then these biomarkers can be helpful for diagnosis, stratification, and monitoring the clinical course. Although some biomarkers have already been incorporated into daily clinical practice, continuous review of their performance is necessary to ensure the safety of clinical decisions based on their results. The importance of the field of biomarkers is evident when analyzing the identification of new host-response biomarkers and the continuous advances made in the fields of genomics, transcriptomics, proteomics, and metabolomics.

Severe pulmonary infections can encompass various forms like CAP, HAP, VAP, ventilator-associated tracheobronchitis (VAT) and acute exacerbations of chronic bronchitis [1, 2, 49, 50]. Host-response biomarkers, predominantly C-reactive protein (CRP) and procalcitonin (PCT), have been applied to these infections. It is worth noting that these as well as other host-response biomarkers are not specific to pulmonary infections. Although these two biomarkers have limitations related to their sensitivity, specificity, dynamics and interaction with the dysfunction of certain organs, both have repeatedly demonstrated the ability to provide additional information about the infectious processes in the lung and can help improve clinical management [12].

### Prediction of VAP

Currently, sepsis is defined as life-threatening organ dysfunction caused by a dysregulated host response to infection and the latest Surviving Sepsis Campaign recommends screening for sepsis in high-risk patients [51]. For patients admitted to the hospital and ICU, this presents a major challenge as, by the time the patients manifests “life-threatening organ dysfunction”, the infection has been present but undetected for some period of time.

A predictive biomarker should enable early and accurate diagnosis of pulmonary infection preferably before symptoms or organ dysfunction become apparent. There may be difficulties in interpretation, as sequential measures of the biomarkers are required (usually not available in CAP), and regarding the lack of specificity for infection vs sterile inflammatory processes.

Several biomarkers and strategies have been studied for early diagnosis. In the BioVAP study [52], the slope and the maximum delta of CRP during the first 6 days of invasive mechanical ventilation were associated with the risk of developing a VAP. A patient with an average increase of CRP concentration of 1 mg/dl/d from D1 till D6 of mechanical ventilation had 62% greater chance of having VAP when compared to a patient with no CRP increase. None of the other biomarkers analyzed, namely PCT, pro-adrenomedullin (pro-ADM), white cell count, and temperature, were helpful in predicting the development of VAP. Two post hoc studies were performed in the same population assessing soluble urokinase plasminogen activator receptor and pancreatic stone protein, both showing poor performance VAP prediction [53, 54].

Among cytokines, the serum concentration of tumor necrosis factor receptor 1 (TNFRI) and plasminogen activator inhibitor-1 (PAI-1), as well as the slope of PAI-1 and IL-10, could potentially be useful for predicting VAP, 3 days prior to clinical onset [55].

Soluble triggering receptor expressed by myeloid cells-1 (sTREM-1) or on the myeloid surface from BAL fluid has been evaluated for VAP diagnosis, showing insufficient accuracy to be implemented as a diagnostic tool [56]. However, a combination of seven biomarkers in BAL fluid and serum, the so-called Bioscore (BALF/blood ratio monocyte surface TREM-1 and monocyte surface CD11b, BALF sTREM-1, IL-8 and IL-1β, and serum CRP and IL-6) correctly identified 88.9% of VAP cases and 100% of non-VAP cases [57] but study replication is still lacking.

A promising line of biomarkers under development are tests that can measure the host response to various stimuli. Sepsis can have different responses, including those that develop hyperinflammation, but also immunosuppression, or a combination of both. This dysregulated response could be diagnosed before signs or symptoms are present by stimulating the immune system. In a recent clinical study, patients with a decreased response in CCL17 to interferon gamma-1b developed HAP [58]. Low expression of HLA-DR on the surface of monocytes was associated with the development of nosocomial infections in patients with septic shock [59].

We have presented the value of different biomarkers or combination of biomarkers in infection prediction, but this approach needs refinement and extension to a comprehensive panel of markers to encompass the complexity of immune responses. Omics, being detection of whole classes of molecules such as proteins (proteome) and metabolites (metabolome), could be used to identify molecular fingerprints related to host/pathogen interaction that may be useful in prediction, diagnosis and prognosis [60].

### Initial assessment of CAP and HAP/VAP

Infection is characterized by a host immune response to damaging or invasive microbial growth [61], and therefore profiling this host response can help identify infection. However, this remains challenging for two major reasons. First, the immune response to sterile and infectious stimuli, initiated by pathogen or damage-associated molecular patterns (PAMPs and DAMPs respectively) [62], uses highly conserved and overlapping pathways [61]. Second, responses to infection are most intense at the site of infection and thus compartmentalized and may not be fully reflected in the blood [63].

- Blood-based biomarkers

As blood has the advantage of sampling almost all tissue beds, and is readily accessible, most host-response biomarkers are blood-based. Table 2 sets out the features of the available tests and those in development. For protein-based biomarkers, notably CRP and PCT, a single determination has modest diagnostic performance in infection and does not reliably distinguish between bacterial and viral infection [12]. These tests may have a helpful role in shortening the duration of antimicrobials in recovering patients [12, 64], or in withholding or withdrawing antibiotics in patients with a low probability of infection. The tests based on parsimonious assays of gene transcription show promise [61] in prediction [65] and detection of infection [66] and distinguishing causative organisms [67]. However, none of these are specific for pulmonary infection.

b) Pulmonary biomarkers

**Table 2:** Host-response biomarkers used in diagnosis of pulmonary infections

Test	Nature of test	Diagnostic performance	Notable features
C-reactive protein	Acute phase protein measured in blood	Moderate sensitivity (~ 80%) for pulmonary infection at time onset, low specificity (~ 60%) Does not effectively discriminate between viral and bacterial infections	Slow to peak (36–48 h), half-life 19 h, false negatives in acute liver failure, IL-6 blockade
Procalcitonin	Hormokine, measured in blood	Low sensitivity (~ 50%) for pulmonary infection, low-moderate specificity (~ 60–70%). Does not effectively discriminate between viral and bacterial infections	Peaks around 24 h, half-life > 24 h, false negatives in early infection and compartmentalized infections, false positives in renal failure, pancreatitis, burns, and some tumors (medullary thyroid carcinoma, lung cancer)
Gene transcription	Various combinations of gene transcripts, ranging from 2 to 29 genes	Limited data in respiratory infections specifically, high sensitivity (~ 80–99%) although specificity ranges from 30–50%. Some combinations show promise in distinguishing bacterial and viral infections	Not yet commercially available, observational data suggest moderate to good diagnostic performance but impact on patient management or outcomes uncertain
Alveolar cytokines	Interleukin 1 beta (IL-1β), CXCL8 have most consistent evidence for use	High sensitivity (90–95%) but low specificity (~ 40–60%)	Requires broncho-alveolar lavage to obtain, no difference in plasma cytokines, no impact on antimicrobial therapy in randomised trial evaluation
Alveolar differential cell count	Percentage of neutrophils from bronchoalveolar lavage	High sensitivity (90–96%) but very low specificity (~ 30%)	Requires broncho-alveolar lavage to obtain, no demonstration of impact on patient management or outcomes
In-vivo alveolar microscopy	Detection of bacteria and neutrophils by fluorescent probes via alveolar fiber imaging (via bronchoscope)	Proof of concept stage, no diagnostic performance established	Diagnostic performance under evaluation, experimental technique and some way from routine availability
Exhaled volatile organic compounds	Detection of volatile organic compounds derived from microorganisms and host cells	Near-patient, real-time devices in development, remain at proof of concept stage	Potential for real-time, continuous monitoring. Existing strategies show modest diagnostic performance

The lungs are readily accessible to diagnostic sampling, especially in invasively ventilated patients. The intense inflammatory response to infection results in elevated alveolar cytokine levels [68] and infiltration of inflammatory cells, most notably neutrophils [69]. Although alveolar cytokines are highly sensitive for pneumonia (Table 2), they lack specificity as other sterile forms of lung injury can lead to similar degrees of elevation [68]. When tested as a rule-out test, alveolar cytokines, though highly specific, did not alter antibiotic use [70]. Although lung fluid cytology and neutrophil counts have not proven sufficient to diagnose pneumonia [69], recent innovations in in-vivo imaging of bacteria and neutrophils show considerable promise [71] and are entering multi-center testing soon (Table 2). Less invasively, the identification of volatile organic compounds in the exhaled air of ventilated patients may be useful in diagnosing respiratory infections and discriminating between infected and colonized patients. However, these non-invasive techniques remain in the research and developmental stage and so far its performance is poor [60].

### Assessment of response to antibiotics

Serum biomarkers have emerged as a tool for monitoring the response to therapy in patients with respiratory infections, serving as surrogate markers for clinical course. Physicians commonly use the kinetics of biomarkers and other variables, including temperature, chest X-rays, white cell count, markers of organ dysfunction (such as creatinine or platelets), over the course of the disease, to assess individual patient prognosis, thus impacting on clinical decision-making and influencing therapeutic strategies.

Reliable evidence collected in recent years demonstrates an intimate association between specific biomarker signatures and adverse clinical outcomes, enabling the predictive enrichment of mortality risk rates. The kinetics of CRP-ratio, relative to the day of infection diagnosis, and the identification of four CRP-ratio patterns have demonstrated utility in the assessment of response to therapy in severe CAP [72] with this ratio unaffected by intercurrent glucocorticoid therapy [73]. Others have reproduced these findings highlighting the value of CRP and PCT kinetics in severe CAP [74, 75]. Similarly, CRP-ratio and its kinetics were also shown to be helpful in VAP [12, 76, 77]. These signature profiles demonstrate significant discriminative power in predicting response to antibiotic therapy and clinical outcomes. Moreover, they have gained recognition in the guidelines of major societies, and their incorporation is advocated as adjunctive tools of patient care [2].

Biomarker-guided antibiotic therapy algorithms have also garnered increased interest, due to their potential to help clinicians reduce antibiotic therapy duration. Two recent meta-analyses concluded that CRP and PCT-guided protocols may significantly improve antibiotic management with significant positive effects on clinical outcomes in hospitalized patients [78, 79]. These biomarker-guided strategies can be incorporated into algorithms including clinical course and duration of therapy, in a multimodal approach strategy [12].

### Antibiotic stewardship

The association between CRP behavior and the clinical response to therapy among patients with pneumonia has been well demonstrated in observational studies [72, 80]. In severe CAP, a CRP concentration exceeding 50% of the initial level after 5 days of antibiotic therapy is indicative of a poor outcome. Additionally, CRP levels higher than 100 mg/dL at the onset of therapy of patients with severe CAP have also been correlated with adverse outcomes [81]. Therefore, CRP was hypothesized as a potential marker to help clinicians tailor antibiotic therapy for hospitalized patients with bacterial infections. To date, only three single-center RCTs have been conducted to test this hypothesis, and showed that CRP-guided strategy can safely reduce the duration of antibiotic therapy [79].

The other commonly available biomarker, PCT, has undergone more extensive studies in protocols of biomarker-guided antibiotic therapy in critically ill patients (at least N = 16 RCT), including pneumonia. Taken together, the results of these studies show that using PCT-guided strategies allows for less antibiotic exposure without harm to patients [82]. However, despite these favorable results, PCT is considerably more expensive and less widely available than CRP, limiting its use in low and middle-income settings. Additionally, PCT is more prone to false-positive results [12]. The prognostic role of other biomarkers in CAP, measured in blood or respiratory secretions, has been tested with variable results [83]. However, unlike CRP and PCT, these molecules have not been assessed as host-response biomarkers in rigorously conducted studies of biomarker-guided antibiotic therapy.

The decision on the safest moment to complete antibiotic therapy in patients with severe pneumonia is complex and requires the collection of numerous clinical, laboratory, and microbiologic information. Therefore, adding a biomarker without a clear definition of its role may prove counterproductive. Hopefully, using digital tools (e.g., smartphone apps, clinical decision support systems) and machine learning-based analyses may aid in integrating these molecules into clinical decisions. This could facilitate the implementation of antibiotic therapy protocols on a broader scale, allowing for more accurate and customized choices at the bedside. These strategies remain in the research and development stage [84].

### Shortcomings of CRP and PCT

Serum CRP, the paradigm of the acute-phase protein, is solely synthesized by the liver in response largely to interleukin 6. Its concentration starts to rise 4–6 h after an inflammatory insult, it doubles every 8 h, and it peaks around 36 to 50 h [85]. The CRP concentration depends only on the intensity of the stimulus and on the rate of synthesis. CRP presents a first-order elimination kinetics with a half-life of 19 h that it is not influenced by underlying disease or therapy except the therapies directed to the primary inflammatory insult [86]. Although being exclusively synthesized in the liver, CRP levels are not influenced by the presence of cirrhosis [87] but in patients with fulminant hepatic failure its level is very low [88]. Besides, acute kidney injury and renal replacement therapy have no effect on CRP level [89, 90]. In the management of critically ill patients, it is important to know that CRP levels in infected patients is not influenced by immunosuppression (either steroids or neutropenia) [73, 91].

Procalcitonin is a prohormone, the precursor of calcitonin, that is classified as an hormokine, since it has properties of hormone and cytokine. PCT is synthesized in virtually all organs and macrophages in response to inflammatory stimuli [92]. Its concentration starts to increase within 3–4 h, peaking around 24 h, and presents a half-life of 22 to 35 h. Since PCT synthesis is not dependent of liver function, its concentration is not affected by cirrhosis nor fulminant hepatic failure [87]. However, since PCT is a small molecule, acute kidney injury is associated with an increase in PCT levels and on the opposite renal replacement therapy with decrease in its concentration making the use of PCT in these patients difficult to interpret [89, 93]. Finally, steroids do not influence PCT levels but in neutropenia there is a higher risk of false negatives.

### Cost-effectiveness

The cost associated with the tests are an important factor to assess its availability. Concerning CRP, the immunoturbidometric assays are reliable, stable, reproducible, have a rapid turnaround time, and are cheap (< 4€ in Europe), with an adequate limit of detection (0.3 to 5 mg/L) for infection management. The high-sensitivity assay of CRP is much more expensive, but it is not necessary in this context. For the measurement of PCT, only the immunoassay based on a Time-Resolved Amplified Cryptate Emission (TRACE) technology presents an acceptable limit of detection (0.06 ng/mL) that is useful for clinical decision-making at the bedside, but at a high cost (€15 in Europe). The TRACE test is also reliable, stable, reproducible, have a rapid turnaround time.

Probably, the first study evaluating the cost-effectiveness of PCT was the ProCAP study [94] showing that the cost of antibiotics plus PCT measurement was significantly higher in the intervention arm. However, the cost of PCT measurement decreased overtime and a recent systematic review and meta-analysis showed that monitoring of PCT was cost saving [95]. These findings have been challenged by studies with real world data [96, 97] showing that PCT monitoring was associated with potential increase in antibiotic days and LOS. CRP measurement is cheaper than PCT, but its cost-effectiveness has not been evaluated systematically [98].

## Conclusions

**Fig. 1:**
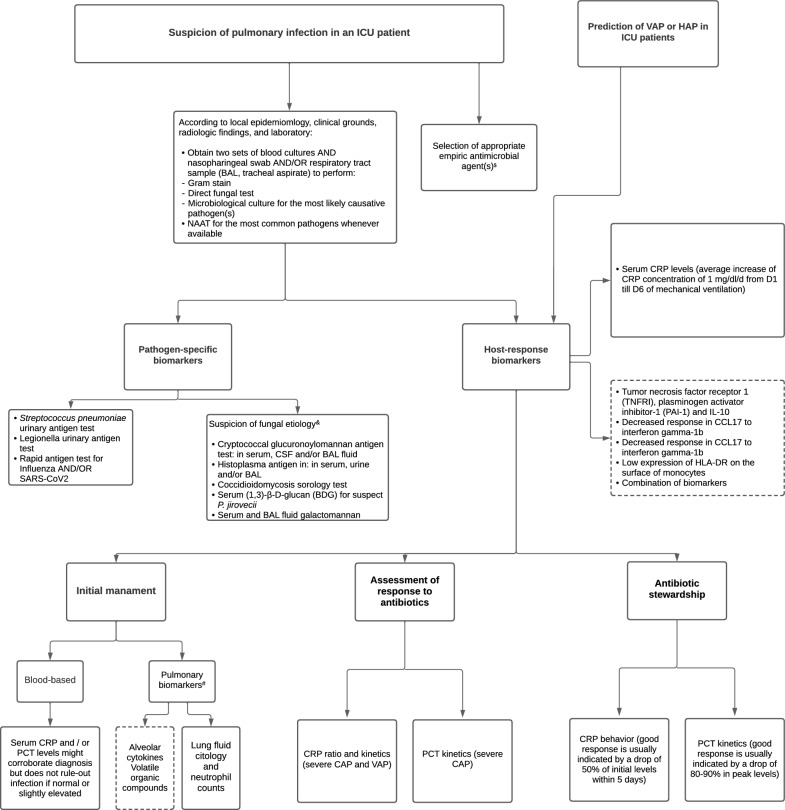
Clinician’s guide to use pathogen-specific and host-response in severe respiratory infection. 1. Refer to the text for details on accuracy of biomarkers, specifics of their indication and limitations; 2. Dashed line indicates experimental biomarkers, not yet incorporated into clinical practice; 3. Bacterial and fungal (especially Aspergillus) pulmonary infection can occur as a complication of primary viral infection (e.g., Influenza, COVID-19); $The antibiotic therapy must be started within 1 h in patients with sepsis and, particulary, in those with septic shock. De-scalation of antibiotics should be made whenever possible after 2–3 days of therapy, based on initial laboratory test results and clinical information."; & These etiologies are more common among imunnocompromised patients (HIV, transplant, use of immunosupressant drugs, among others); #Specially in invasively ventilated patients. *NAAT* nucleic acid amplification test; *CSF* cerebrospinal fluid, *CRP* C-reatvie protein, *PCT* procalcitonin

Biomarkers may have an adjunct role in diagnosing pulmonary infections in critically ill patients, and in tailoring antimicrobial treatment (Fig. 1 and ESM). Pathogen-specific biomarkers are currently used to identify several bacterial, mycobacterial, viral, and fungal pulmonary infections, such as *Streptococcus pneumoniae*, *Legionella spp*., *Mycobacterium tuberculosis*, SARS-CoV-2, Influenza, *Pneumocystis jirovecii*, Cryptococcus spp., and *Histoplasma capsulatum*. Serum and BAL galactomannan are supportive markers to diagnose invasive pulmonary aspergillosis in at-risk ICU patients, and BDG increases the diagnostic yield of pneumocystis pneumonia. Host-response biomarkers, such as CRP and PCT, may be useful in diagnosing bacterial pulmonary infections. However, a single determination has moderate diagnostic performance for infection and do not reliably distinguish between bacterial and viral infection. The value of pulmonary biomarkers should further be evaluated as serial determinations. The use of host-response biomarker-guided strategies allow for less antibiotic exposure and side effects, while maintaining patients’ safety and treatment efficacy. Thus, the appropriate use of accurate biomarkers may benefit both the bedside patient care by enhancing the diagnostic precision, as well as the antibiotic stewardship by safely reducing the utilization of unnecessary antimicrobials.

### Supplementary Information

Supplementary Material 1.

## Data Availability

Not applicable.

## Abbreviations

AIDS: Acquired immunodeficiency syndrome
BAL: Bronchoalveolar lavage
BDG: (1,3)-β-D-glucan
CAP: Community-acquired pneumonia
CRAG: Cryptococcal glucuronoxylomannan antigen
CRP: C-reactive protein
CSF: Cerebrospinal fluid
DAMP: Damage associated molecular patterns
EIA: Enzyme immunoassay
HAP: Hospital-acquired pneumonia
HIV: Human immunodeficiency virus
ICU: Intensive care unit
LAM: Lipoarabinomannan
NAAT: Nucleic acid amplification technology
OTR: Organ transplant recipients
PAI-1: Plasminogen activator inhibitor-1
PAMP: Pathogen associated molecular patterns
PJP: *Pneumocystis jirovecii* Pneumonia
PCR: Polymerase chain reaction
PCT: Procalcitonin
RSV: Respiratory syncytial virus
sTREM-1: Soluble triggering receptor expressed by myeloid cells-1
TB: Tuberculosis
TNFRI: Tumor necrosis factor receptor 1
VAP: Ventilator-associated pneumonia
VAT: Ventilator-associated tracheobronchitis
WHO: World Health Organization
