# Supplementary material for: Biomarkers in pulmonary infections: a clinical approach
Source: Ann Intensive Care. 2024 Jul 17;14:113. doi: 10.1186/s13613-024-01323-0 (PMC11254884; doi:10.1186/s13613-024-01323-0)
Supplement: Supplementary file 1 — Supplementary Material 1. [file 13613_2024_1323_MOESM1_ESM.docx]

AOIC-D-24-00247 R1

Pedro Póvoa, Luís Coelho, José Pedro Cidade, Adrian Ceccato, Andrew Conway Morris, Jorge Salluh, Vandack Nobre, Saad Nseir, Ignacio Martin-Loeches, Thiago Lisboa, Paula Ramirez, Anahita Rouzé, Daniel A Sweeney, Andre C Kalil

Annal of Intensive Care

Electronic Supplemental Material

Clinical vignettes where biomarkers, pathogen-specific biomarkers, and host-response biomarkers, can add crucial information on top of clinical, laboratory and radiologic data in the management of severe pulmonary infection.

**Clinical Scenario #1**

A 56-year-old male patient, heavy smoker, was brought to the emergency department (ED) from home by his wife because he was complaining of fever, productive cough, some diarrhoea in the previous 2 days and today he became confused. At admission, he presented with altered mental status (Glasgow Coma Score, GCS – 13), temperature 38.5°C, heart rate 102 bpm, blood pressure 92/55mmHg, respiratory rate 33 bpm, SpO_2_ on room air 86%. On physical examination he presented with central cyanosis, no meningeal signs, lungs showed bilateral rales and crackles (more on the left), abdomen was painful and distended, without tenderness; the remainder of the physical examination was normal. Chest X-rays showed mid and lower zone patchy consolidations (more on the left) and no pleural effusion. Blood tests showed leucocytosis (14,400/μL), normal platelet count, elevated creatinine (1.3 mg/dL; one month before was 0.9 mg/dL) and C-reactive protein (CRP) of 32 mg/dL and procalcitonin of 2.1 ng/dL.

The clinical suspicion was for severe community-acquired pneumonia (CAP) in an immunocompetent patient and according to current guidelines the patient should start antibiotic therapy with combination therapy with a beta-lactam plus a macrolide.

Additionally, some pathogen-specific biomarker tests were performed, namely influenza, SARS CoV2, pneumococcus and Legionella. Since the Legionella urinary antigen test was positive the antibiotic therapy was changed to levofloxacin. It is also important to add that in patients with suspicion of CAP, a CRP at admission >25mg/dL is associated with *L. pneumophila* pneumonia [1]. Finally, such a finding should be notified to the local health authorities to exclude the presence of an outbreak.

**Clinical Scenario #2**

A 61-year-old man, with ischemic heart disease, was admitted to the ED with fever (38.6°C), cough, purulent sputum, and right pleuritic chest pain. At admission, he was tachypneic (respiratory rate 36 bpm), tachycardic (hear rate 102 bpm) and hypotensive (85/40 mmHg), with a capillary refill time of 4 sec and oliguric. Arterial blood gas presented a moderate hypoxemia (PaO2 55 mmHg at room air) and lactate was 4.1 mmol/l. Chest X-rays revealed a consolidation in the right lower lobe. Blood tests showed leukocytosis (28,000/μL), creatinine 3.7 mg/dl, CRP 16.3 mg/dL and PCT 2.5 ng/dL. Pneumococcal urinary antigen test was positive, PCR for influenza and SARS CoV2 (nasal swab) were negative. Sputum and blood cultures were performed. Antibiotic therapy with ceftriaxone and azithromycin was prescribed. He was admitted to ICU, the clinical condition deteriorated further, and he was started on invasive mechanical ventilation (with FiO2 of 60-70%), vasopressor support and renal replacement therapy (RRT).

On the 3rd day, he had no fever and the hemodynamic condition improved. Biomarkers showed a decrease: CRP 12.1 mg/dL (75% of initial value) and PCT 0.75 ng/dL (30% of initial value). However, the patient still needed mechanical ventilation and RRT. At day 4, penicillin-sensitive Streptococcus pneumoniae was identified from sputum and blood cultures. Antibiotic therapy was de-escalated to penicillin.

On the 7th day, the fever recurred (39°C) with new hemodynamic deterioration. The Chest X-rays presented a new pleural effusion on the right hemithorax confirmed by the CT scan. Biomarkers presented an increase of CRP to 24 mg/dL (biphasic response pattern) and PCT 0.3 ng/dL. Diagnostic thoracentesis was performed with drainage of purulent pleural fluid and cytochemical examination compatible with empyema. A thoracic drain was placed with a drainage of 1000 ml of purulent pleural fluid.

On the 9th day, the patient was clinically better, with no fever and improvement of oxygenation (PaO2/FiO2 280). Chest X-rays showed a marked reduction of pleural effusion and a well-positioned chest drainage with a daily drainage of 120 ml of serous pleural fluid. Mechanical ventilation and vasopressor support were progressively weaned. Blood tests presented a normal leukocyte count (8.300/μL) and CRP reduced to 8.1 mg/dL and PCT to 0.1 ng/dL. The bacteriological culture of the pleural fluid was negative.

On the 12th day, the patient was stable, the chest drain was removed, and he was transferred to the ward the day after. Antibiotics were stopped on the 15th day of therapy.

References

[1] Garcia Vazquez E, Martinez JA, Mensa J *et al.* C-reactive protein levels in community-acquired pneumonia. Eur Respir J 2003; 21:702-705.
